# A Framework to Determine the Extent to Which Regional Primary Healthcare Organisations Are Comprehensive or Selective in Their Approach

**DOI:** 10.34172/ijhpm.2020.182

**Published:** 2020-10-05

**Authors:** Sara Javanparast, Fran Baum, Anna Ziersch, Toby Freeman

**Affiliations:** ^1^College of Medicine and Public Health, Flinders University, Adelaide, SA, Australia.; ^2^Southgate Institute for Health, Society and Equity, Flinders University, Adelaide, SA, Australia.

**Keywords:** Comprehensive Primary Healthcare, Assessment Framework, Regional Planning, Australia

## Abstract

**Background:** There is an increasing emphasis on the importance of comprehensive primary healthcare (CPHC) in improving population health and health equity. There is, therefore, a need for a practical means to determine how comprehensive regional primary healthcare organisations (RPHCOs) are in their approach. This paper proposes a framework to provide such a means. The framework is then applied to assess the comprehensiveness of Australian RPHCOs.

**Methods:** Drawing on a narrative review of the broader literature on CPHC versus selective primary healthcare (SPHC) and examples of international models of RPHCOs, we developed a framework consisting of the key criteria and a continuum from comprehensive to selective interventions. We applied this framework to Australian RPHCOs using data from the review of their planning documents, and survey and interviews with executive staff, managers, and board members. We used a spidergram as a means to visualise how comprehensive they are against each of these criteria, to provide a practical way of presenting the assessment and an easy way to compare progress over time.

**Results: **Key criteria for comprehensiveness included (1) focus on population health; (2) focus on equity of access and outcomes; (3) community participation and control; (4) integration within the broader health system; (5) inter-sectoral collaboration; and (6) local responsiveness. An examination of Australian RPHCOs using the framework suggests their approach is far from comprehensive and has become more selective over time.

**Conclusion:** The framework and spidergram offer a practical means of gauging and presenting the comprehensiveness of RPHCOs, and to identify gaps in comprehensiveness, and changes over time.

## Background

**Key Messages**

Implications for policy makers

- Regional structures of primary healthcare (PHC) are established in many countries including Australia to facilitate regional planning, address community needs, and coordinate and integrate PHC services in a defined geographical area and it is important for policy-makers to ensure their approach is comprehensive.
- Comprehensive primary healthcare (CPHC) is important to improve population health and achieve equity in health access and outcomes. A means is required to judge the extent to which regional primary healthcare organisations (RPHCOs) are comprehensive or selective in their approach.
- Determining how comprehensive RPHCOs are will assist PHC planners, managers and other stakeholders to identify gaps, plan interventions to improve comprehensiveness, and monitor changes over time.
- While our approach was derived from an empirical study in Australia it promises to have value for other countries.

Implications for the public

Comprehensive primary healthcare (CPHC) is proven to be effective in improving population health and people’s access to a range of treatment, prevention and health promotion services. It also places an emphasis on addressing social factors that impact the health of individuals and populations. An examination of national and regional PHC organisations is important to determine the extent to which they are able to provide comprehensive primary healthcare (PHC) to different population groups. This paper reports on an approach to assess the comprehensiveness of regional primary healthcare organisations (RPHCOs) and its application in Australia. It may assist to identify gaps in PHC, changes over time, and areas that need improvement to achieve equity in health access and outcomes.

Stronger primary healthcare (PHC) systems are recognised as leading to better performance in terms of population health and equitable health outcomes,^1^ and for providing an infrastructure for improved integration of care to prevent and treat chronic and complex health conditions.^2^ The Alma Ata Declaration in 1978 defined *comprehensive *primary healthcare (CPHC) as a philosophy of health and a multi-disciplinary service model underpinned by values of equity and engagement, that focuses on a range of activities including treatment, prevention and health promotion.^3^ It also places an emphasis on addressing social determinants that impact the health of individuals and populations.^4^ In contrast, *selective* primary healthcare (SPHC), proposed soon after the Alma Ata conference,^5^ prioritises the fight against selected diseases based on cost-effective medical interventions. The tension between comprehensive and selective PHC has long been noted globally and still remains central in health policy agendas.^6^ More recently, the importance of CPHC was re-affirmed in the World Health Organization (WHO) Astana Declaration on PHC^7^ and as ‘the programmatic engine for Universal Health Coverage in most contexts.’^8^ Evidence from many countries confirms that implementation of CPHC has been patchy, with a more selective approach overtaking the original vision of PHC.^9^ In Australia, CPHC has been mainly realised in community health centres and Aboriginal Community Controlled Health Organisations which have a long history of practice based on a social model of health and equity.^10^

 Decentralised and sub-national PHC structures have been recommended by the WHO, underpinned by the notion that locally-operated PHC can engage more effectively in localised and collaborative health planning and decision-making, and promote local autonomy.^11^ In many low- and middle-income countries ‘district health systems’ have been used as a vehicle for the implementation of PHC in local communities^12^ and to deal with context-specific challenges and priorities such as PHC information systems.^13^ In several high-income countries, regional primary healthcare organisations (RPHCOs) have been established to facilitate regional planning, address community needs, and coordinate and integrate PHC services in a defined geographical area.^14^ The establishment of RPHCOs in Australia goes back to the establishment of Divisions of General Practice in 1992. Divisions were proposed as a means to promote the coordination of local PHC services while maintaining medical autonomy.^15^ In 2011, a network of 61 Federally-funded Medicare Locals (MLs) evolved from the Divisions to develop regional needs assessment and planning, and to implement, monitor and evaluate strategies within a defined geographical boundary.^16^ In 2015, as a result of a change of government, MLs were replaced by new structures, 31 primary health networks (PHNs), covering a larger catchment area and continuing to play a role in identifying needs, planning and care coordination.^17^ Nevertheless, the Federal Government identifies the health priorities that are planned for and implemented by PHNs. While MLs provided services and only very limited commissioning, the main role of PHNs is to commission rather than provide services.

 Examples of regional PHC structures in other high-income countries include Primary Healthcare Trusts in England (now transformed to clinical commissioning groups),^18^ New Zealand Primary Healthcare Organisations,^19^ Ontario Local Health Integration Networks,^20^ and Scotland Community Health Partnerships.^21^ These models explicitly emphasise a regional approach to PHC and population health which puts responsiveness to community needs, addressing the needs of local populations, making services locally and culturally-sensitive, and multidisciplinary care at the centre of service planning and implementation.^22^

###  Evaluation Frameworks and Tools

 Several evaluation frameworks and tools have been developed to measure PHC service performance. For example WHO’s ‘primary care evaluation framework’ has been used in a number of countries, and provides a structured approach to PHC assessment based on specific aspects of the health system, such as governance, funding and resource generation, as well as factors that characterise good PHC, including access, comprehensiveness, coordination and continuity.^23^ Other tools have been developed that are more centred on PHC service’s performance through provider or client perspectives, as well as PHC domains including access, comprehensiveness, and coordination.^24^ Other evaluation studies have typically focused on individual components of CPHC such as community participation or equity rather than the assessment of the PHC approach of a whole system,^25^ or have a disease-centred focus such as prescribing medicine or referrals.^26^ Existing tools mainly assess one element of PHC or its service performance. The WHO evaluation framework, although a more comprehensive one, is not designed to assess comprehensive versus selective PHC, and lacks a focus on regional structures of PHC. Despite the specific role that RPHCOs play in identifying local needs, regional planning and service integration, and the importance of organisational governance in local planning and partnership, there are no frameworks in the literature to guide PHC planners, managers and other stakeholders to examine comprehensiveness of RPHCOs, and to identify gaps, plan interventions, and monitor changes over time. This paper reports on research which enabled us to apply the criteria we identified above from the literature to assess and visualise (using a spidergram) the comprehensiveness of RPHCOs (as distinct from specific services).

 Based on a narrative review^27^ of the indexed and available grey literature (including policy documents) from a number of high-income countries, six dimensions distinguish comprehensive from selective PHC in the context of RPHCOs: focus on population health; focus on equity of access and outcomes; community participation and control; integration with the broader health system; intersectoral collaboration and local responsiveness. For the purpose of this paper, we looked at the RPHCOs primarily from high-income countries with similarities to Australia such as New Zealand, the United kingdom, and Canada.

###  Focus on Population Health – the Extent to Which PHC Planning and Programs Are Based on the Health of Whole Population and Incorporate a Continuum of Curative, Rehabilitative, Preventive and Health Promotion Services

 A population health approach is at the core of CPHC and takes a population rather than individual orientation to health and well-being.^28,29^ In the case of RPHCOs, at one extreme are organisations with an emphasis on individual care and risk factors – for example in the United Kingdom^30,31^ and New Zealand^32,33^ where services lean heavily towards curative approaches. On the other hand, Local Health Integration Networks in Ontario (more recently replaced by Ontario Health) had a stronger emphasis on community-based services to support population health.^34^

###  Focus on Equity of Access and Outcomes – Attention to Health Equity Through Action on Social Determinants of Health

 Literature on CPHC^9,29^ including the report of WHO Commission on Social Determinants of Health^4^ suggests that although access to high quality care is crucial, to be most comprehensive, PHC must be accompanied by coordinated actions on wider determinants of health. Our review of international RPHCOs found that in most cases a selective approach is taken to address inequity through strategies to improve access to medical services. In contrast, a comprehensive approach has a greater focus on equity of health outcomes through advocacy and action on social determinants of health.^35^

###  Community Participation and Control – Level of Community Engagement and Transfer of Power to Communities to Take Control of Their Health and Health Decision-Making

 Our narrative review indicated that CPHC widely acknowledges the importance of community participation and control in health planning and implementation. Power and control over health decisions and building capacity of local people were highlighted in the Alma Ata Declaration on PHC^3^ and in the literature that compares comprehensive versus selective PHC.^36,37^ Our review of RPHCOs revealed a continuum of community engagement ranging from tokenistic approaches where communities are only consulted to identify needs to more comprehensive approaches to community empowerment and control guided by community engagement frameworks and mechanisms.^31,38,39^

###  Integration Within The Broader Health System – Level of Structural/Functional Vertical Integration With the Broader Health System and Collaboration With Local or Regional Health Organisations, Secondary and Tertiary Health System Via Formal Mechanisms

 An important element of CPHC is to collaborate with and integrate with the broader health system to avoid fragmentation of services and improve the provision of continuous and comprehensive care.^40^ RPHCOs were varied in the extent to which they fostered integration While some countries such as the United Kingdom showed ambiguity about relationships with other PHC partners and the broader health system,^30^ a formal linkage between RPHCOs and other sectors of health system including state divisions of health was mandated in Ontario.^41^

###  Inter-sectoral Collaboration – Level of Collaboration With Non-health Sectors in PHC Planning

 Literature on CPHC emphasises collaboration with sectors outside of health as an integral component of CPHC and as a way to address social determinants of health and achieve health equity.^42^ Many models of RPHCOs identified inter-sectoral collaboration as one of their key goals or used various strategies through formal or informal structures to include stakeholders in PHC planning and implementation. For example, linking with government and non-government organisations, and social services to protect and promote the health of local populations were explicitly mentioned in both the New Zealand and Ontario’s government documents.^32,34^ There is, however, less evidence on how the policy goals have been supported and implemented to improve inter-sectoral collaboration in PHC planning and actions.

###  Local Responsiveness – Level of Flexibility in Funding for Locally Tailored Programs and Organisational Authority in Responding to Local Needs

 Being responsive to local needs is one of the key elements of CPHC and an important rationale for establishing regional and de-centralised PHC structures.^11^ Resource allocation formulas for distributing the funding, flexibility in how the funding is used, and local authority are all factors contributing to organisational capacity to plan and implement locally-tailored programs.^43^ Despite variability in funding models and levels of autonomy in different RPHCOs, most RPHCO models have shown that the national government typically retain control of strategic policy and priority setting, with little flexibility for RPHCOs to respond the local needs.^44^

## Methods

###  Development of the Framework

 We turned the key criteria identified from the literature into a tabular framework to demonstrate a continuum from selective to comprehensive PHC (Table 1). The criteria were presented as five points on a continuum, from a more selective approach to PHC at one end (1), moving towards a comprehensive PHC approach at the other end (5). Definitions were added under each criteria (for anchors 1, 3, and 5) to clearly distinguish key focus areas and provide examples of activities and services across the continuum from selective to comprehensive PHC. Ratings 2 and 4 are intended to be given when an organisation falls midway between two descriptors (scores 1, 3 and 5). The framework intends to be used as a means to triangulate information from a variety of sources and reach a holistic judgement about the extent to which a RPHCO is selective or comprehensive.

**Table 1:** Framework to Determine the Extent to Which RPHCOs Are Comprehensive or Selective in Their Approach

**Key Elements**	**Continuum From Selective To Comprehensive PHC**				
	**(Selective PHC)** **1**	** 2 **	**3**	**4**	**(Comprehensive PHC)** **5**
Focus on population health	***Individual care***	**− − − − − − −− − − − −− − − − − − − − − − − − − − −> **			***Population health***
	Focus on individuals and curative care; medical interventions; disease-specific care	Main focus on curative care, and behavioral and lifestyle interventions; some attention on population health and prevention (mainly screening and immunisation)			Continuum of curative, rehabilitative, preventive and health promotion services in planning and priority setting; strong focus on the health of the whole population
Focus on equity of access and outcomes	***No focus on equity***	**− − − − − − −− − − − −− − − − − − − − − − − − − − −> **			***Equity of access and outcomes***
	No focus on equity; focus on disease specific strategies without attention to equity of access or outcomes	Interventions to facilitate equity of access; targeting specific population groups in need; Some evidence of collecting population data on social determinants of health			Focus on equity and social determinants of health; attention to equity of outcomes in the whole population through action on the social determinants of health
Community participation and control	***No community participation***	**− − − − − − −− − − − −− − − − − − − − − − − − − − −> **			***Community controlled***
	No community engagement or control in planning and decision-making	Some degree of community engagement mainly in identifying needs; limited engagement of communities in decision-making and priority setting; limited transfer of power to communities			Community controlled; community representation in organisational decision-making structure (eg, board membership)
Integration within the broader health system	***Working in silo***	**− − − − − − −− − − − −− − − − − − − − − − − − − − −> **			***Integration within the broader health system***
	No collaboration with the broader health system in governance, health planning, resource allocation and program implementation	Some degree of vertical collaboration with broader health system eg, data sharing; informal mechanisms for collaboration eg, regular meetings			Structural/functional vertical integration with the broader health system; strong collaboration with local or regional health organisations, secondary and tertiary health system via formal mechanisms
Inter-sectoral collaboration	***No collaboration outside health sector***	**− − − − − − −− − − − −− − − − − − − − − − − − − − −> **			***Strong inter-sectoral collaboration***
	No collaboration with non-health sectors eg, local government, housing, employment and education	Some degree of collaboration with non-health sectors; informal relationships eg, occasional meetings on specific local projects			Strong collaboration with non-health sectors: joint planning and priority setting; formal mechanisms for collaborative work eg, memorandum of understanding, board membership
Local responsiveness	***Central management and control***	**− − − − − − −− − − − −− − − − − − − − − − − − − − −> **			***Flexible and local response***
	Central funding allocation and priorities; no pool of flexible funding	Some degree of local funding flexibility and priority setting, with locally tailored programs			High level of flexible funding for locally tailored programs; organisational authority in responding to local needs

Abbreviations: RPHCOs, regional primary healthcare organisations; PHC, primary healthcare.

###  Application of the Framework

 Data from Australian RPHCOs (both previous MLs and current PHNs) were collected as part of a 4-year project funded by the National Health and Medical Research Council (2014-2018). Data collection methods include:

####  Document Review

 Guidelines and documents produced by the Australian Federal government for the MLs and PHNS were reviewed for aims, priority areas and funding models. Publicly available documents including needs assessments, activity plans and annual reports from 61 MLs (2012-2013, 2013-2014) and 31 PHNs (2015-2016, 2016-2017) were obtained from their websites. Collated documents were then transferred to QSR NVivo software and coded based on CPHC criteria. The research team regularly met to discuss contents fitting under each code. Two members of the research team double coded documents from a number of MLs and PHNs to ensure rigour.

####  Online Survey

 Two rounds of online surveys were conducted with executive staff, managers and board and council members in MLs (September-November 2014) and PHNs (July-October 2016) to explore PHC priorities and approaches to regional planning and programs. The ML survey instrument was adapted for PHNs and included comparable items on engagement strategies, organizational efforts, capacity and effectiveness in population health planning, equity, and addressing social determinants of health (Supplementary file 1). For both surveys, the study information and links to the online survey were sent to the chief executive officer (CEOs) for completion and distribution amongst relevant staff and board/council members. We used the Dillman method^45^ to increase the response rate by sending in-advance notification to CEOs, followed by three email reminders in 3 week intervals. We received 210 responses from 52 MLs (85% of MLs) and 66 responses from 17 PHNs (55% of PHNs). Simple descriptive statistics were used to analyse survey data in SPSS software to describe PHC performance against a number of PHC characteristics including equity, engagement and partnership.

####  Telephone Interviews

 Semi-structured telephone interviews were conducted with 50 ML senior executives and board members (October 2014-January 2015). ML survey respondents were offered an option to provide their contact for follow up interviews, with 106 (50%) indicated their willingness to participate. The final selection of interview participants was based on their seniority and involvement in population health planning, and their geographical location (eg, both urban and rural regions). A different approach was used for the PHN interviews. Participants were purposively selected from 6 PHNs that were willing to participate and located in different states and territories as well as from rural and metro areas. Invitations sent through CEOs and of a total of 82 people invited, 55 people (67%) agreed to participate in a telephone interview. Interviews explored planning for population health, partnerships, community engagement, organisational capacity and funding models that facilitated or inhibited the implementation of a CPHC approach (Supplementary file 1). Interviews were audio-recorded, transcribed and de-identified before being transferred to QSR NVivo software and analysed thematically. A coding framework was developed including themes from the literature and those emerging from the documents and interview data and regularly discussed by the research team. Eight ML and four PHN interviews were double coded by team members and discussed for consistency and rigour.

 Data from different sources were triangulated and analysed using the continuum of the PHC criteria to score both MLs and PHNs against each criteria presented in the evaluation framework (Table 1). The scores given to MLs and PHNs for each criteria were discussed in the research team until a consensus was reached.

 We transferred the CPHC criteria and their five indicators into a spidergram by plotting these indicators on a continuum. Spidergrams are often used to visualize and undertake a rapid assessment of health programs and interventions.^46,47^ The agreed value for each criteria was then charted in the spidergram to visualize the extent to which Australian RPHCOs have incorporated comprehensive PHC.

## Results

 In this section we use data from our study to assess the extent to which RPHCOs were comprehensive or selective in their approach to PHC against each criteria. We also compare the two forms of RPHCOs supported by the Australian Government (the MLs with the PHNs) to determine the extent to which the criteria enable us to compare the two.

###  Focus on Population Health

 The development of population health plans based on regional needs assessment has been mandated for Australian RPHCOs. Despite variations between individual organisations, findings from document reviews, and survey and interview data found that overall the concept of population health has been more towards a selective approach where preventive and health promotion services are largely overlooked in planning, programs and funding allocation. The lack of clarity about the role of RPHCOs in population health and primary prevention was consistently shown across data sources and is reflected in this quote: “*It’s [population health] the area that we’ve probably struggled with the most. We’ve been less focused on developing population-wide approaches within our region. Going right back to the strategic objectives, I don’t think it’s ever been entirely clear to us what is our role in terms of primary prevention or health promotion” *(ML, interview).

 Comparative analysis of data over time indicated a further shift towards SPHC with more emphasis on individual care and medical interventions with the advent of PHNs. Survey data showed that MLs devoted significantly more effort to health promotion (t(252) = 4.2, *P* < .001), and reported greater capacity for health promotion than the current PHNs (t(257) = 3.4, *P* = .001). One PHN executive noted “*compared to previous MLs, it appears to me that the focus in the PHNs is less primary preventive than they are secondary preventive. We’re basing it on disease.”* Nevertheless, prevention and health promotion activities were mainly centred on screening programs, immunisation and lifestyle interventions.

###  Focus on Equity

 The Australian policy priorities, funding models, and activity plans mainly focus on the equity of access (to medical services) and targeting population groups. Despite the collection of population data on social determinants of health such as housing, employment, and education as revealed in needs assessment documents, survey and interview data consistently showed little policy support, organisational capacity, or authority to act on social determinants of health to improve health equity: “*What we do find about social determinants, is that we get knocked back from the federal Department whenever we put up something that they see as falling too outside of the health umbrella” *(PHN, CEO-1). The RPHCO restructure from MLs to PHNs increased the focus on access to medical services rather than action on social determinants of health. Survey data showed that MLs devoted significantly more effort to acting on SDH (t(254) = 4.7, *P* < .001), and reported greater capacity to act on SDH (t(256) = 2.5, *P* = .013) than PHNs. Consistently, interview data confirmed a further move away from social determinants approach in PHNs. A typical comment was “*I would say that the focus on equity and social determinants has subsided under this government compared to when we were first established as MLs” *(PHN, Deputy CEO).

###  Community Participation and Control

 We found a low degree of community engagement in regional PHC planning and activities. In general, the establishment of RPHCOs in Australia provided an opportunity to include community members in governance. The mandate for the current PHNs to include community councils in their governance structure was a positive step towards strengthening community inputs. Nevertheless, in response to a survey question ‘to what extent community members are involved in decision-making process’ PHNs reported that community members were involved to a significantly lesser extent than the MLs did (t(270) = 3.28, *P* = .001). Interview respondents also supported the lack of meaningful engagement with local communities: A PHN program manager reported: *“we as an organisation don’t understand that community engagement is not an activity, that it is a way of working. And that engagement requires us to be transparent, then in constant dialogue and taking account of the needs and situations of the people. That it’s not just about if we go out and run forums and tell them what’s expected and what they need to do, that we have engaged and therefore they will deliver.” *Organisational boards, as the main decision-making structure, had limited representation from community members in both MLs and PHNs.

###  Integration With the Broader Health System

 We found a growing emphasis on partnership with state/territory departments of health and their regional structures, which oversee tertiary systems in Australia. Data collected from different sources indicated an emphasis on vertical collaboration in both MLs and PHNs. For example, approximately 91% of ML staff and 87% of PHN staff who completed the survey reported ‘somewhat’ or ‘very’ effective engagement with actors in state departments of health or their regional structures respectively. Mechanisms for partnership with departments of health such as memorandum of understanding, board representation and regular meetings at executive levels and examples of joint planning and programs and sharing funding were evident in the majority of annual reports and supported by interview data. PHNs policy guidelines explicitly emphasised partnership with state/regional health structures (local health networks –LHNs).^17^ In transition from MLs to PHNs, the Australian Department of Health attempted to align PHNs’ geographical boundaries with the LHNs to facilitate collaboration. One interview participants stated: ‘*We have a perfect alignment of the PHN and state department of health, if you like, in respect of opportunity, and that is widely recognised by everybody and we have excellent working relationships with our state government’ *(PHN, CEO-2).

*“They [LHNs] were more than actively engaged. And so in some respects it was joint planning really. There was a lot of resource that went into that and a lot of capacity and a lot of sophistication” *(ML, Board member).

###  Inter-sectoral Collaboration

 Across the data we found very limited inter-sectoral collaboration for both MLs and PHNs. Levels of engagement with sectors outside health such as housing, schools and councils of social services were rated the lowest in surveys. Survey data showed that MLs devoted significantly more effort to acting on SDH (t(254) = 4.7, *P* < .001), and reported greater capacity to act on SDH (t(256) = 2.5, *P* = .013) through intersectoral collaboration than PHNs. This was supported by interview data “*I understand one of the differences from MLs to PHN is that it’s a bit of shift back to being working with GPs only’* (PHN, manager), Another interviewee stated: “W*hen you’re talking about social determinants, like housing and education and jobs, it is about a multi-pronged approach with those sectors. I think those collaborative type of initiatives are not clearly funded” *(PHN, CEO-3).Little evidence was also found in PHNs annual reports demonstrating engagement with other sectors in PHC planning and program implementation.

###  Local Responsiveness

 A lack of flexibility in funding as an inhibiting factor to plan and implement locally tailored programs was a common theme emerging from different data sources. Centrally allocated funding for specific programs that were prioritised by the Federal government were a major feature of both MLs and PHNs. The transition in the PHC structure was associated with a reduction in flexible funding preventing PHNs responding to local needs as noted below: “*PHNs have a mandate to do a comprehensive healthcare needs assessment. Great. They go and do that. But if the funding conditions that are set out by the Commonwealth don’t support meeting those needs, it puts them in a really awkward position. They know what the issues are but actually they don’t have the flexibility to use the funds in a way that will enable meeting what those community needs and priorities are” *(PHN, senior executive).

 The findings from the Australian PHC study for each of the identified criteria is summarised in Table 2.

**Table 2:** Assessment of Australian Regional PHC Organisations to Determine How Comprehensive They Are

**CPHC Criteria**	**Study of Australian PHC Organisations**			
	**MLs (2011-2014) **	**Score**	**PHNs (2015-Current) **	**Score**
Focus on population health	Mandate to develop population health plans for the region Focus on access to medical services Some evidence of prevention and health promotion activities	2.5	Mandate to develop ‘work activity plans’ in specific areas identified by the federal government eg, mental health, drug and alcohol, integrated care Focus on access to medical services through commissioning processes Little evidence of prevention and health promotion activities	1.5
Focus on equity of access and outcomes	Strong focus on equity of access to medical services Targeting selected population groups in need Collection of population data on equity and social determinants of health Some examples of actions on social determinants of health	3	Commissioning organisation with a sole focus on improved access to medical services Very little evidence of action on social determinants of health	1.5
Community participation and control	Community consultations for needs assessments Community advisory groups in some MLs Low level of community representation on board No community feedback mechanisms	2.5	Community consultations for needs assessment Mandatory structure of community advisory council on governance Low level of community representation on board No community feedback mechanisms	2.5
Integration within the broader health system	Good evidence of working with state level LHNs and tertiary care Some evidence of joint planning and resource sharing	2	Greater emphasis on working with LHNs and tertiary care (efforts to align boundaries) Some evidence of joint planning and resource sharing	3
Inter-sectoral collaboration	Some limited evidence of working with non-health sectors such as local government, housing and transport	3	Little evidence of partnership with non-health sectors to address social determinants of health	1
Local responsiveness	Centrally managed programs, but with some evidence of funding flexibility and capacity for locally tailored programs	3	Little evidence of funding flexibility, reduction in flexible funding pool Centrally managed programs Little capacity for locally tailored programs	1.5

Abbreviations: CPHC, comprehensive primary healthcare; PHC, primary healthcare; MLs, Medicare Locals; LHNs, local health networks; PHNs, primary health networks.

 The ratings we allocated to MLs and PHNs for each criteria are shown in the spidergram in Figure. The use of a spidergram assists in visualising the level of comprehensiveness and to easily compare RPHCOs.

**Figure:**
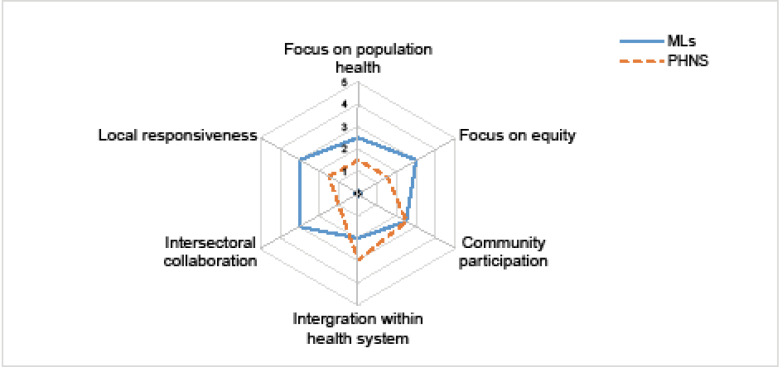
Comprehensiveness Ratings for Australian PHC Organisations (MLs, 2011-2015, PHNs, 2015-current). Abbreviations: PHC, primary healthcare; MLs, Medicare Locals; PHNs, primary health networks.

## Discussion

 Regional structures of PHC in many countries are established to facilitate planning for population health and equity in a defined geographical area, coordinate and integrate local activities, and to assess and respond to local community’s needs.^11^ An assessment of how comprehensive or selective they are in their approach to PHC is important to enable health planners to determine ways in which to maximise the comprehensiveness of PHC, though the means to guide PHC planners to do such assessments is lacking. The CPHC criteria identified in this study is underpinned by the Alma Ata definition of CPHC and sought to include those criteria required for regional structures of PHC to be comprehensive. The ability to assess the form of PHC by applying these criteria will assist national and regional health planners and policy-makers to further their understanding of PHC comprehensiveness, to examine the extent to which RPHCOs incorporate CPHC elements, and to identify gaps and areas for improvement.

 There are points of strengths in our process of developing CPHC criteria and the assessment of comprehensiveness in Australian RPHCOs. Firstly, the framework captures key principal criteria of CPHC. Our review of broader literature on CPHC and RPHCOs supplemented by examples of RPHCOs in a number of countries similar to Australia assisted in identifying areas of focus and their alignment with the Alma Ata definition of CPHC. An examination of national and regional policies, guidelines and activities in selected countries provided an opportunity to define a continuum (from selective to comprehensive) under each criteria.

 In applying the framework into Australian RPHCOS, we had access to a wide range of qualitative and quantitative data that we had collected as part of a larger study. This provided us a strong evidence base on which to score Australian RPHCOs against our CPHC criteria. For future use of this framework, a standard data collection method (including both quantitative and qualitative data) at different levels of administration (policy-makers, managers and providers) as well as recipients of services is highly recommended to ensure sufficient data are available to make a thorough assessment.

 Furthermore, our 4-year study witnessed a change of Australian government and subsequently a major change in the structure and policy direction of RPHCOs. Although this added to the complexity of the assessment, it provided a chance to compare two forms of RPHCOs. Evidence suggests that support for comprehensiveness in the policy and operational environment is critical to services being able to deliver CPHC.^48,49^

 Unfortunately, we have found that in Australia the ability of RPHCOs to pursue and champion CPHC has been severely constrained by a neoliberal approach to PHC policy and implementation,^50^ and this is a common barrier to CPHC globally.^51^ PHC continues to be contested, with the comprehensive vision of PHC vying with the more selective, technical approach to PHC that much more closely aligns with neoliberalism.^50^ Thus, this framework may be valuable in articulating comprehensiveness or lack of comprehensiveness in the mission, goals, and activities of RPHCOs, and changes over time (towards or further away from comprehensiveness). The impact of external factors influencing CPHC in Australia is explained elsewhere.^50,52^ Using the proposed framework in countries with different regulatory and policy systems to Australia will be helpful to examine how external factors may influence comprehensiveness of PHC systems. It also helps to refine the framework to make it more context-specific.

 Finally, the use of a spidergram created a quick and easy way to visualise the level of comprehensiveness in Australian RPHCOs and to identify areas that need further attention, and also illustrate the comparison between two different organisations (MLs and PHNs) and track the progress over time. Spidergrams are widely used as a means to visualize and compare elements of health programs and interventions.^46,47^ Such a visual tool will be helpful to health planners seeking to encourage a more comprehensive PHC system.

 The process had some limitations. We focused our review of international RPHCOs primarily from a number of high-income countries because of their similarities to Australia. This, however, limits the generalisability and usefulness of the framework in different settings. We acknowledge that in many low- and middle-income countries as well as other high-income countries strong developments have occurred in PHC. Although this framework has the potential for application in countries with regional structures of PHC, its application may yield further insights and refinements. Future studies reviewing CPHC criteria in other country settings, for example high income countries with a different regulatory environment to Australia as well as low- and middle-income countries where RPHCOs are generally different in scope and nature to those in high income countries, and the application of the proposed framework to assess their comprehensiveness is highly recommended.

 Another limitation relates to the process of scoring Australian RPHCOs against each criteria identified in the framework. We acknowledge that there were/are variations within 61 MLs and 31 PHNs concerning the implementation of CPHC that goes back to numerous organisational factors, local context, and actors within each organisation who influence action.^52^ This may be the case in other settings too. Giving a score to the whole organisation under each criteria does not capture these variations. Lastly, our data did not include perspectives from clients or service users who used services within the regions covered by the organisations, mainly because MLs and PHNs were not primarily providers of PHC services in their regions. We also acknowledge that PHNs have made advances since we finalised our data collection in 2016. The research provides a snapshot of PHNs approach at one particular period of time in their early development to illustrate the use of the framework. Re-assessment of PHNs will provide information about any changes that may have occurred since the completion of this study. This issue may be applicable to other countries which face changes in PHC policy and practice over time.

## Conclusion

 Determining the comprehensiveness of RPHCOs is vital given the tendency for PHC to become selective. The framework we have developed will be useful to health policy-makers, planners and managers in determining the extent to which their RPHCOs are comprehensive or selective in their approach. It enables identification of areas in which improvements needs to be made. The use of a spidergram model provides a user-friendly means of mapping change over time or to compare two organisations. Further application of this framework will assist in the global movement to increase the comprehensiveness of PHC planning and service provision and so result in improved population health and equity.

## Ethical issues

 Ethics approval was granted by the Flinders University Social and Behavioural Research Ethics Committee (SBREC).

## Competing interests

 Authors declare that they have no competing interests.

## Authors’ contributions

 SJ undertook the review of international models of PHC organisations, contributed to data collection and analysis of the Australian study, and led the writing of the paper. FB, AZ, and TB contributed to data analysis and interpretation, commented on drafts of the paper and approved the final draft for submission.

## Funding

 The Australian study was funded by the National Health and Medical Research Council (NHMRC Application number 1064194).

## Authors’ affiliations

^1^College of Medicine and Public Health, Flinders University, Adelaide, SA, Australia. ^2^Southgate Institute for Health, Society and Equity, Flinders University, Adelaide, SA, Australia.

## Supplementary files


Supplementary file 1. Medicare Locals and Primary Health Networks Survey instruments and Interview Schedules.
