# Supplementary material for: A Framework to Determine the Extent to Which Regional Primary Healthcare Organisations Are Comprehensive or Selective in Their Approach
Source: Int J Health Policy Manag. 2020 Oct 5;11(4):479–88. doi: 10.34172/ijhpm.2020.182 (PMC9309948; doi:10.34172/ijhpm.2020.182)
Supplement: Supplementary file 1 — Medicare Locals and Primary Health Networks Survey instruments and Interview Schedules. [file ijhpm-11-479-s001.pdf]

Supplementary file 1. Medicare Locals and Primary Health Networks Survey Instruments and Interview Schedules

Medicare Locals Survey

This survey is part of a larger study funded by the NH&MRC and is being conducted by the Southgate Institute for Health, Society and Equity, Flinders University. The **Participant Information Sheet** provides further information about the overall project and the investigator team.

The survey aims to explore your perspective on Medicare Locals' contribution to the function and achievements of primary health care, and factors facilitating or constraining population health planning. We appreciate that Medicare Locals will be replaced by Primary Health Networks after July 2015. This survey provides a great opportunity to examine the role that Medicare Locals have played in improving care coordination, health equity and access, their achievements to date, and lessons to be learned for the operation of Primary Health Networks in the future.

Your participation is invaluable in providing a picture of Medicare Locals across Australia and the role that they have played in improving access and equity, care coordination, and population health planning.

We are particularly interested in how Medicare Locals have identified and addressed the health needs of three population groups:

- *Aboriginal and Torres Strait Islander people*
- *New migrants/refugees*
- *People with mental illnesses.*

The information you provide will be anonymous and no personal information will be collected. While we ask the name of your Medicare Local and your position this is only to assist us categorising findings based on the location and participants' role and cross-referencing with key documents. We will present the data in an aggregated format that does not allow any participant to be identifiable.

The survey will take approximately 20 minutes to complete. You are of course free to withdraw from the survey at any time or refuse to answer any questions**.**

As a thank you for your time completing the survey and sharing your experience with us, we offer a **$30 voucher.** You will have the choice of an iTunes or Coles/Myer gift voucher (both

redeemable online) or an Oxfam shop gift voucher at the completion of the survey. You will be redirected to a page at the end of the survey that allows you to claim your voucher.

Consent
I have read the information provided above and in the Participant Information Sheet and consent to participate in this survey

□ I Agree

1. **Name of your Medicare Local:** (Data will be presented in an aggregated format that does not identify your Medicare Local)

2. **Which State/Territory is your Medicare Local located in?**
   ○ ACT
   ○ NSW
   ○ NT
   ○ QLD
   ○ SA
   ○ TAS
   ○ VIC
   ○ WA

3. **What is your position at your Medicare Local?**
   ○ CEO/Deputy CEO
   ○ Senior Executive
   ○ Program Manager
   ○ Chair of board
   ○ Board member
   ○ Other (specify)……………

4. **What do you see as the biggest achievements of your Medicare Local since its establishment?**

1.<br><br>2.<br><br>3.

5. **How do you rate the effectiveness of your Medicare Local's engagement with the following groups?**

	Very ineffective	Somewhat ineffective	Neutral	Somewhat effective	Very effective	Have not engaged with this group
General practitioners	○	○	○	○	○	○
Practice nurses	○	○	○	○	○	○
Allied health providers	○	○	○	○	○	○
State government primary health care services	○	○	○	○	○	○
Local hospitals	○	○	○	○	○	○
State/Territory department of health (including LHNs)	○	○	○	○	○	○
Health consumer organisations	○	○	○	○	○	○
Aboriginal community controlled health services	○	○	○	○	○	○
Aboriginal community organisations	○	○	○	○	○	○
New migrants/refugees health organisations	○	○	○	○	○	○
Public mental health services	○	○	○	○	○	○
Private mental health services	○	○	○	○	○	○
Mental health NGOs	○	○	○	○	○	○
Local government	○	○	○	○	○	○
Local housing providers	○	○	○	○	○	○
Local schools	○	○	○	○	○	○
Council of social service	○	○	○	○	○	○

Please provide any comments here.

6. **To what extent has your Medicare Local involved community members in key decision making processes?**

Not at all	To a small extent	To a moderate extent	To a large extent	To a very large extent
○	○	○	○	○

Can you please give an example of community members' involvement?

7. **What successes has your Medicare Local had in engaging with *<u>Aboriginal and Torres Strait Islander community members</u>*?**

8. **Is there anything that your Medicare Local could have done better to engage more effectively with *<u>Aboriginal and Torres Strait Islander community members</u>*?**

9. **What successes has your Medicare Local had in engaging with *<u>new migrant/refugee community members</u>*?**

10. **Is there anything that your Medicare Local could have done better to engage more effectively with *<u>new migrant/refugee community members</u>*?**

11. **What successes has your Medicare Local had in engaging with *<u>people with mental illness</u>*?**

12. Is there anything that your Medicare Local could have done better to engage more effectively with *people with mental illness*?

13. What successes has your Medicare Local had in engaging with *people living in socio-economically disadvantaged circumstances?*

14. Is there anything that your Medicare Local could have done better to engage more effectively with *people living in socio-economically disadvantaged circumstances?*

15. Please indicate below how much EFFORT your Medicare Local has invested in the following activities since its establishment?

	**Very low**	**Low**	**Medium**	**High**	**Very high**
Provision of clinical services	○	○	○	○	○
PHC service coordination (eg, referral pathways, linking with local health providers)	○	○	○	○	○
Chronic disease management coordination	○	○	○	○	○
Disease prevention (eg, immunisation, screening, smoking cessation)	○	○	○	○	○
Health promotion (services/programs that enable people to stay well and increase their control over the determinants of health to improve their health)	○	○	○	○	○
Acting on social determinants of health (eg, housing, transport, etc)	○	○	○	○	○
Developing population health plans	○	○	○	○	○

Please provide comments on your answers

16. How do you rate the CAPACITY of your Medicare Local to do the following activities?

	Very low	Low	Medium	High	Very high
Provision of clinical services	○	○	○	○	○
PHC service coordination (e.g referral pathways, linking with local health providers)	○	○	○	○	○
Chronic disease management coordination	○	○	○	○	○
Disease prevention (eg, immunisation, screening, smoking cessation)	○	○	○	○	○
Health promotion (services & programs that enable people to stay well and increase their control over the determinants of health to improve their health)	○	○	○	○	○
Acting on social determinants of health (eg, housing, transport, etc)	○	○	○	○	○
Developing population health plan	○	○	○	○	○

Please provide comments on your answers

17. To what extent do you see the following factors as obstacles for your Medicare Local doing population health activities (including disease prevention, health promotion and social determinants of health)?

	Not at all	To a small extent	To a moderate extent	To a large extent	To a very large extent
Inadequate funding	○	○	○	○	○
Lack of workforce skills	○	○	○	○	○
Inadequate physical and IT infrastructure within the Medicare Locals	○	○	○	○	○
Difficulty in engaging with partners	○	○	○	○	○
Lack of boundary alignment with State/Territory	○	○	○	○	○

department of health regions (including LHNs)					
Lack of performance indicators for population health activities	○	○	○	○	○
Policy context not supportive of population health	○	○	○	○	○
Health system prioritising clinical care	○	○	○	○	○
Others (please specify) ………………………………..	○	○	○	○	○

Please provide comments on your answer

18. How do you rate the support your Medicare Local received from the following organisations for population health activities (including disease prevention, health promotion and social determinants of health)?

	**Very poor**	**Poor**	**Fair**	**Good**	**Excellent**
Australian Government Department of Health	○	○	○	○	○
Australian Medicare Local Alliance (AMLA)	○	○	○	○	○

Please provide comment

19. How appropriate is the *geographical size* of your Medicare Local for your population health activities (including disease prevention, health promotion and social determinants of health) ?

**Much too small**	**Too small**	**Appropriate size**	**Too big**	**Much Too big**
○	○	○	○	○

Please provide comment

20. How appropriate is the *population size* of your Medicare Local for your population health activities (including disease prevention, health promotion and social determinants of health)?

Much too small	Too small	Appropriate size	Too big	Much Too big
○	○	○	○	○

Please provide comment

21. What are the three most important lessons to be learned from the operation of your Medicare Local that may be useful for the new Primary Health Networks?

1.

2.

3.

22. What do you think will be the strengths of the proposed Primary Health Networks?

23. What do you think will be the weaknesses of the proposed Primary Health Networks?

24. Please provide any other comments

Thank you for completing the survey. We appreciate your input.

Supplementary to this survey, we would like to interview a sample of survey participants to further improve our understanding of the role that Medicare Locals have played in population health, equity to access, and care integration. The interviews will be conducted by one of our senior researchers, will take no longer than 30 minutes, will be done face-to-face or over the telephone and at a time that is convenient for you.

You will now be redirected to a second page to select your $30 voucher and to provide your contact details if you would like to participate in an interview session.

Please be assured that your contact details will not be linked to your responses in the present survey.

To receive your gift voucher:
Please provide your name and postal address to send the $30 voucher.
Name:

Address 1:

City/Town:

State/Province:

ZIP/Postal Code:

Please select the gift voucher you prefer
○ iTunes
○ Oxfam
○ Coles/Myer
○ No, thank you

If you are willing to be interviewed please provide us with your name and contact number:

Name:

Phone Number:

Primary Health Networks Survey

This survey is part of a study funded by the NH&MRC and is being conducted by the Southgate Institute for Health, Society and Equity, Flinders University. The **Participant Information Sheet** provides further information about the overall project and the investigator team.

The survey aims to explore your perspective on PHNs' contribution to the function of primary health care, and factors facilitating or constraining population health planning.

Many PHNs have been established by the amalgamation of previous Medicare Locals with boundary changes while some are totally new organisations. We are interested in your views on how the new Primary Health Networks facilitate population health planning and primary health care engagement and coordination. Your participation is invaluable in providing a picture of PHNs across Australia and the role that they play in improving access and equity, care coordination, and population health planning.

This survey is adapted from a similar survey we conducted with Medicare Locals in late 2014. The findings from this survey will enable us to compare and contrast some aspects of PHC planning in PHNs with the previous Medicare Locals.

We are particularly interested in how PHNs have identified and addressed the health needs of three population groups:

- *Aboriginal and Torres Strait Islander people*
- *New migrants/refugees*
- *People with mental illness*
- *People living in low socio-economic conditions*

The information you provide will be anonymous and no personal identifying information will be collected. We do ask the name of your PHN and your position but this is only to assist us categorising findings based on the location and participants' role. We will present the data in an aggregated format that will not allow any participant to be identifiable.

The survey will take approximately 20 minutes to complete. You are of course free to withdraw from the survey at any time or refuse to answer any questions.

As a thank you for your time completing the survey and sharing your experience with us, we offer a **$30 voucher.** You will have the choice of an iTunes or Coles/Myer gift voucher (both redeemable online) or an Oxfam shop gift voucher at the completion of the survey. You will be redirected to a page at the end of the survey that allows you to claim your voucher.

Consent

I have read the information provided above and in the Participant Information Sheet and consent to participate in this survey

□ I Agree

25. What is the name of your Primary Health Network (PHN): (Data will be presented in an aggregated format that does not identify your PHN)

26. Which State/Territory is your PHN located in?

○ ACT
○ NSW
○ NT
○ QLD
○ SA
○ TAS
○ VIC
○ WA

27. What is your position at this PHN?

○ CEO/Deputy CEO
○ Executive
○ Program Manager
○ Chair/member of board
○ Chair/member of clinical council
○ Chair/member of community advisory committee
○ Other (please specify)……………

28. Were you employed by/worked for a previous ML?

○ **Yes**
○ **No**

29. If yes, was the ML you were employed by/worked for:

○ **in the same region as this PHN**
○ **in different region as this PHN**

30. What was the name of the ML/s you were employed by/worked for:

Governance and decision making

31. To what extent do you think the Board composition of your PHN is appropriate?

Not at all	To a small extent	To a moderate extent	To a large extent	To a very large extent
○	○	○	○	○

Please explain your response

32. To what extent do you think the advice from the community councils has influenced decision making?

Not at all	To a small extent	To a moderate extent	To a large extent	To a very large extent
○	○	○	○	○

33. To what extent do you think the advice from the clinical councils has influenced decision making?

Not at all	To a small extent	To a moderate extent	To a large extent	To a very large extent
○	○	○	○	○

34. To what extent has your PHN involved community members (other than the Community Council members) in key decision making processes?

Not at all	To a small extent	To a moderate extent	To a large extent	To a very large extent
○	○	○	○	○

Please provide any comments here

Engagement and Partnerships

35. How do you rate the effectiveness of your PHN's engagement with the following groups since your PHN was established?

	Very ineffective	Somewhat ineffective	Neutral	Somewhat effective	Very effective	Have not engaged with this group
General practitioners	○	○	○	○	○	○

Practice nurses	○	○	○	○	○	○
Allied health providers	○	○	○	○	○	○
State government primary health care services	○	○	○	○	○	○
Local hospitals	○	○	○	○	○	○
State/Territory department of health (including LHNs)	○	○	○	○	○	○
Health consumer organisations	○	○	○	○	○	○
Aboriginal community controlled health services	○	○	○	○	○	○
Aboriginal community organisations	○	○	○	○	○	○
New migrants/refugees health organisations	○	○	○	○	○	○
Public mental health services	○	○	○	○	○	○
Private mental health services	○	○	○	○	○	○
Mental health NGOs	○	○	○	○	○	○
Local government	○	○	○	○	○	○
Local housing providers	○	○	○	○	○	○
Local schools	○	○	○	○	○	○
Council of social service	○	○	○	○	○	○
Private health insurers	○	○	○	○	○	○

Please provide any comments here.

36. To what extent do the requirements of your funding agreement with the Australian Government allow your PHN to meet the needs of your community in terms of the following:

	**Not at all**	**To a small extent**	**To a moderate extent**	**To a large extent**	**To a very large extent**
Population health planning for your region	○	○	○	○	○
Commissioning for gaps in PHC services in your region	○	○	○	○	○

Addressing the social determinants of health	○	○	○	○	○
Planning services for Aboriginal and Torres Strait Islander people	○	○	○	○	○
Planning services for migrants and refugees Planning services	○	○	○	○	○
Planning services for people with mental illness	○	○	○	○	○
Planning services for people from low SES background	○	○	○	○	○

Please provide comments on your answers

37. Please indicate below how much EFFORT your PHN has invested in the following activities since its establishment?

	**Very low**	**Low**	**Medium**	**High**	**Very high**
Developing population health plans	○	○	○	○	○
Provision of clinical services	○	○	○	○	○
Commissioning activities	○	○	○	○	○
PHC service coordination (e.g referral pathways, linking with local health providers)	○	○	○	○	○
Chronic disease management coordination	○	○	○	○	○
Disease prevention (eg, immunisation, screening, smoking cessation)	○	○	○	○	○
Health promotion (services/programs that enable people to stay well and increase their control over the determinants of health to improve their health)	○	○	○	○	○
Acting to influence social determinants of health (eg, housing, transport, etc)	○	○	○	○	○
working with Private Health Insurers	○	○	○	○	○

Please provide comments on your answers

38. How do you rate the CAPACITY of your PHN to do the following activities?

	Very low	Low	Medium	High	Very high
Developing population health plans	○	○	○	○	○
Provision of clinical services	○	○	○	○	○
Commissioning activities	○	○	○	○	○
PHC service coordination (e.g referral pathways, linking with local health providers)	○	○	○	○	○
Chronic disease management coordination	○	○	○	○	○
Disease prevention (eg, immunisation, screening, smoking cessation)	○	○	○	○	○
Health promotion (services & programs that enable people to stay well and increase their control over the determinants of health to improve their health)	○	○	○	○	○
Acting to influence social determinants of health (eg, housing, transport, etc)	○	○	○	○	○
Working with Private Health Insurers	○	○	○	○	○

Please provide comments on your answers

39. To what extent do you see the following factors as obstacles for your PHN doing population health activities (including disease prevention, health promotion and social determinants of health)?

	Not at all	To a small extent	To a moderate extent	To a large extent	To a very large extent
Inadequate funding	○	○	○	○	○
Inflexibility in the use of funding					
Lack of workforce skills	○	○	○	○	○
Insufficient staffing					

Inadequate physical and IT infrastructure within the PHNs	○	○	○	○	○
Difficulty in engaging with partners	○	○	○	○	○
Lack of boundary alignment with State/Territory department of health regions (including LHNs)	○	○	○	○	○
Lack of performance indicators for population health activities	○	○	○	○	○
Policy context not supportive of population health	○	○	○	○	○
Health system prioritising clinical care	○	○	○	○	○
Others (please specify) ………………………………..	○	○	○	○	○

Please provide comments on your answer

40. How do you rate the support your PHN has received from the Australian Government Department of Health for population health activities (including disease prevention, health promotion and social determinants of health)?

	**Very poor**	**Poor**	**Fair**	**Good**	**Excellent**
Australian Government Department of Health	○	○	○	○	○

Please provide comment

41. **How would you rate your PHN's success in addressing the health needs of *<u>Aboriginal and Torres Strait Islander people?</u>***

**Very unsuccessful**	**unsuccessful**	**Neither successful nor unsuccessful**	**Successful**	**Very successful**	**Don't know**
○	○	○	○	○	

42. **If you worked at a ML previously, how would you rate your previous ML's success in addressing the health needs of *__Aboriginal and Torres Strait Islander people?__***

Very unsuccessful	unsuccessful	Neither successful nor unsuccessful	Successful	Very successful	Don't know
○	○	○	○	○	

Please provide your comments here:

43. Can you describe examples of successes that your PHN have had to date in engaging with *__Aboriginal and Torres Strait Islander community members__*? Please write answer in the box.

44. What could your PHN have done better so far to engage more effectively with *__Aboriginal and Torres Strait Islander community members__*? Please write answer in box.

45. **How would you rate your PHN's success in addressing the health needs of *__new migrants and refugees?__***

Very unsuccessful	unsuccessful	Neither successful nor unsuccessful	Successful	Very successful	Don't know
○	○	○	○	○	

46. **If you worked at a ML previously, how would you rate your previous ML's success in addressing the health needs of *__new migrants and refugees?__***

Very unsuccessful	unsuccessful	Neither successful nor unsuccessful	Successful	Very successful	Don't know
○	○	○	○	○	

Please provide your comments here:

47. **Can you describe examples of successes that your PHN have had to date in engaging with *new migrant/refugee community members*? Please write answer in box.**

48. **What could your PHN have done better so far to engage more effectively with *new migrant/refugee community members*? Please write answer in box.**

49. **How would you rate your PHN's success in addressing the health needs of *people with mental illness?***

Very unsuccessful	unsuccessful	Neither successful nor unsuccessful	Successful	Very successful	Don't know
○	○	○	○	○	

50. **If you worked at a ML previously, how would you rate your previous ML's success in addressing the health needs of *people with mental illness?***

Very unsuccessful	unsuccessful	Neither successful nor unsuccessful	Successful	Very successful	Don't know
○	○	○	○	○	

Please provide your comments here:

51. **Can you describe examples of successes that your PHN have had to date in engaging with *people with mental illness*? Please write answer in box.**

52. **What could your PHN have done better so far to engage more effectively with *people with mental illness*? Please write answer in box.**

53. **How would you rate your PHN's success in addressing the health needs of *__people living in socio-economically disadvantaged circumstances?__***

**Very unsuccessful**	**unsuccessful**	**Neither successful nor unsuccessful**	**Successful**	**Very successful**	**Don't know**
○	○	○	○	○	

54. **If you worked at a ML previously, how would you rate your previous ML's success in addressing the health needs of *__people living in socio-economically disadvantaged circumstances?__***

**Very low**	**Low**	**Medium**	**High**	**Very high**	**Don't know**
○	○	○	○	○	

Please provide your comments here:

55. Can you describe examples of successes that your PHN have had to date in engaging with *__people living in socio-economically disadvantaged circumstances?__* Please write answer in box.

56. What could your PHN have done better so far to engage more effectively __with__ *__people living in socio-economically disadvantaged circumstances?__* Please write answer in box.

__Transitions from Medicare Locals to PHNs__

The following questions explore issues in relation to the transitions from MLs to PHNs and their impact on population health planning processes in your PHN. If you are not aware of the transition process for your PHN, please feel free to select the 'Don't know' responses.

57. How do you rate the following aspects of the transition from MLs to PHNs in your PHN?

	**Very poor**	**Poor**	**Fair**	**Good**	**Very good**	**Don't know**
Retention of population health planning skills and expertise						
Maintaining the MLs' momentum for population health planning						
Maintaining partnerships						
Maintaining services to fill identified gaps						
Building capacity in commissioning activities						
Establishing a new governance structure						
Others ………………….						

Please provide any comments here

58. How appropriate is the *geographical size* of your PHN for your population health activities (including disease prevention, health promotion and social determinants of health)?

**Much too small**	**Too small**	**Appropriate size**	**Too big**	**Much Too big**
○	○	○	○	○

How have any changes in the geographical size from previous MLs to your PHN affected population health planning in this PHN?

59. How appropriate is the *population size* of your PHN for your population health activities (including disease prevention, health promotion and social determinants of health)?

Much too small	Too small	Appropriate size	Too big	Much Too big
○	○	○	○	○

How have any changes in the population size from previous MLs to your PHN affected population health planning in this PHN?

60. Please provide any other comments in the box below:

Thank you for completing the survey. We appreciate your input.

You will now be redirected to a second page to select your $30 voucher
Please be assured that your contact details will not be linked to your responses in the present survey.

To receive your gift voucher:
Please provide your name and postal address to send the $30 voucher.
Name:

Address 1:

City/Town:

State/Province:

ZIP/Postal Code:

Please select the gift voucher you prefer
○ iTunes
○ Oxfam
○ Coles/Myer
○ No, thank you

Medicare Local project – Staff interview
Discussion topics

[**Note:** trying to collect stories, asking more open questions and probing examples]

Starting the conversation
What we are interested in this study is your experience, as a senior member of this ML, on population health planning, access and equity, disease prevention and health promotion and social determinants of health, and how your ML managed to address these issues.

- What do you see as the overarching mission of your ML? *[their impression of the mission]*

Keyword: Population health planning

We know that population health planning and needs assessment have been a key part of all MLs functions.

- Can you tell me a bit more about the process your ML followed?

Probe: Things that went well
Things that didn't go well
Capacity, skills and orientation of ML regarding population health approach
Engagement with stakeholders including LHN and their involvement in decision making and priority setting
Enablers and constraints (eg, timelines, funding, other support, population and geographical size)
Performance indicators, measuring progress, evaluation

Keyword: Equity

- How do you think this ML has addressed the issue of equity?

Probe: Discuss measures aimed at equity of access versus equity of outcome
Key strategies used e.g targeting areas or population groups most in need
Examples of improved access and success factors
Things did not go well and why

Keyword: Disease prevention and health promotion

There has been always a tension between planning for clinical work and for broader disease prevention and health promotion activities.

- How has this played out in your ML? What drove the decisions?

Probe: Any discussion around this at management level and its dynamic
Factors influencing decision (funding and resources, policy and political context, workforce orientation and capacity)

Stakeholders' engagement including LHN, community groups and providers
Examples of successful disease prevention and health promotion activities, what did and did not go well and why

Keyword: Social determinants of health

- Has SDoH been on your ML's agenda? Can you tell me how your ML went about addressing SDoH?

Probe: Examples of action addressing SDoH and success factors
Things that didn't go well and why
Level of engagement with non-health organisations, challenges
Enablers and constrains (organisational capacity and orientation, funding support and resources, etc)
How often your executive team discuss SDoH?

Keyword: Funding model

- How do you think the current funding model has facilitated or constrained population health activities including disease prevention and health promotion, and social determinants of health?

Probe: Funding allocation
Amount
Flexibility/autonomy

Keyword: Executive decision making

Now we would like to talk about decision making process in your ML.

- How do you think your executive decision making processes facilitate or constrain population health planning activities?

Probe: Issues that took most of executive time
Issues that were prioritised

Equity Groups

The next part of the interview has a focus on specific groups of population and how your ML went about identifying and meeting their needs. The three groups of interest in our study are Aboriginal and Torres Strait Islanders, new migrants and refugees, and people with mental illness but we can talk about any particular group that your ML has targeted to improve equity.

Keyword: Aboriginal and Torres Strait Islanders

We found from the survey and review of MLs documents that most MLs have Aboriginal health/closing the gap program to improve Aboriginal health.

- Can you tell me a bit more about your programs and what they aimed to achieve?

Probe: Strategies used to identify the needs of Aboriginal and Torres Strait Islander people
Engagement with Aboriginal health services, organisations and community members (eg, Aboriginal staff, Aboriginal representatives in board, advisory groups and committees, etc)
Examples of where the program went well and key success factors, things that did not go well and why
How well the needs of Aboriginal people have been met
Enablers and barriers (funding and resources, workforce capacity, cultural awareness, etc)

Keyword: New migrants and refugees

- Has new migrants and refugees health been a priority area in your region? If not, why? If yes, can you tell a bit about how did your ML go about improving equity and access for this group?

Probe: Strategies used to identify the needs of new migrants and refugees
Engagement with migrant community members (challenges given the diversity in culture and language)
Engagement with migrant services and organisations
Organisational capacity and competency
Examples of programs that went well, success factors
Things that didn't go well and why
How well the needs of migrants and refugees people have been met
Enablers and barriers (funding and resources, workforce capacity, cultural awareness, etc)

Keyword: people with mental illness

Our third group of interest in the study is people with mental illness. We know that most ML have planned/implemented a number of mental health programs.

- How do you think your ML went in addressing the health needs of people with mental health?

Probe: Strategies used to identify the needs of people with mental illness
Engagement with people with mental illness and challenges
Engagement with mental health services (public, private, NGOs)
Organisational capacity and competency
Examples of programs that went well, success factors
Things that didn't go well and why
How well the needs of people with mental health have been met
Enablers and barriers (funding and resources, workforce capacity, etc)

Keyword: people living in low socio-economic status

- How has your ML targeted this group in the planning and programs?

Probe: Strategies used to identify the health and social needs
Engagement with people living in low SES

Examples of programs that went well, success factors
Things that didn't go well and why (addressing SDoH)

Keyword: Other groups

- Are there any other specific groups in the region that you worked with (eg, disability, carers, elderly, homelessness, etc)? How did you select these groups?

Probe: Strategies used to identify their needs
Engagement with the group members
Engagement with relevant health services and organisations (public, private, NGOs)
Examples of programs that went well, success factors
Where a program did not go well and why

Probe (if not covered): For the equity groups and population programs that we just discussed were these informed by the population health planning and needs assessment?

Keyword: The role of a national body in coordinating regional PHC organisations

- What do you see the role of a national body in supporting regional primary health care organisations? How did AMLA provide support to your Medicare Local? What could have been done differently?

Keyword: Divisions of General practice in the past/future Primary Health Networks

- Did you have any experience of previous divisions of general practice? If yes, how is the ML compared with the division?

- What do you see as the key opportunities that would be offered by PHNs?

- What do you see the risks of changing the structure of regional PHC organisations from MLs to PHNs? What do you think would be lost/gained?

Final question

- Just as a final question, could you tell me what has been the best for you about working in ML?

PHN interviews
Discussion points

The questions was tailored for each of the six participating PHN to clearly reflect their transition from ML, board composition and activities.

- Transition from MLs to PHNs
  - Process
  - Factors facilitating or constraining the transition
  - Workforce issues
  - Changes in funding and service models
  - Any other issues

- Governance: structure and members
  - Board composition and expertise
  - Local Health District involvement - benefits
  - Private Health Insurers involvement – benefits/disadvantages
  - Clinical councils – composition, role, decision making, experience in population health planning
  - Community advisory committee – composition, role, decision making power

- Population health planning – process, capacity, partnership
  - Skills and competency
  - Clinical vs population health activities (equity, health promotion and social determinants of health)
  - Monitoring and evaluation
  - Partnership with stakeholders
  - Community engagement

- Equity groups
  - Aboriginal and Torres Strait Islander – plan, workforce, engagement
  - People with mental illnesses – plan, workforce, engagement
  - New migrants and refugees – plan, workforce, engagement
